# Chinese EFL learners’ processing of English binomials: the role of interlexical and intralexical factors

**DOI:** 10.3389/fpsyg.2024.1425856

**Published:** 2024-09-09

**Authors:** Zhuo Chen, Nan Fang

**Affiliations:** ^1^Department of General Courses, Guangzhou Panyu Polytechnic, Guangzhou, China; ^2^School of Foreign Studies, Shaoguan University, Shaoguan, China

**Keywords:** L2 binomial processing, congruency, frequency, predictability, reversibility

## Abstract

Binomials have been relatively understudied compared to other types of multiword expressions (MWEs) in second language research, such as collocations and idioms. This study investigated English as a Foreign Language (EFL) learners’ processing of English binomials and how it is influenced by interlexical factors (L1-L2 congruency and L1-lexicalization) and intralexical factors (word and binomial frequency, binomial reversibility, and binomial predictability). Forty Chinese EFL learners participated in a phrase acceptability judgment task of 64 target binomials (16 congruent L1-lexicalized, 16 congruent L1-nonlexicalized, and 32 incongruent) and 64 non-binomial controls. Results revealed that learners experienced difficulty judging the formulaicity of binomials. They processed binomial stimuli significantly faster than non-binomial baselines, demonstrating a binomial phrase effect. They also processed L1-L2 congruent items faster and more accurately than incongruent items, showing a robust congruency effect. The congruent items which are lexicalized in the L1 showed further processing advantage than the non-lexicalized items, indicating a graded congruency effect. Moreover, binomial reversibility and binomial predictability (measured with cloze probability) also showed significant effects. These findings highlight the need to distinguish and investigate different types of congruency, explore appropriate measures for MWE predictability, and to examine binomials focusing on their unique features.

## 1 Introduction

Multiword expressions (MWEs) or formulaic sequences (Wray, 2002) have received considerable research interest for the last several decades for their ubiquity in actual language use (Siyanova-Chanturia and Pellicer-Sanchez, 2019), their importance for language fluency and general proficiency (Boers, 2020), and also for their profound implications for theories of language acquisition (Christiansen and Arnon, 2017).

Binomial is an important type of MWEs (Mollin, 2014). It is “a coordinated pair of linguistic units of the same word class which show some semantic relation,” such as *men and women*, *up and down*, and *odds and ends* (Kopaczyk and Sauer, 2017, p. 3). Binomials possess several unique characteristics that make them “an ideal test case for models of the processing and mental representation of multi-word units” (Mollin, 2014, pp. 16–17). First, they have a distinctive “configuration” (Siyanova-Chanturia and Janssen, 2018, p. 2016) or “template” (Carrol and Conklin, 2020, p. 116), i.e., their fixed and conspicuous structure of *A and B*. Besides, binomials show different degrees of reversibility (Mollin, 2014): some binomials are used exclusively in one order, with the reversed form mostly infelicitous (e.g., *by and large*), while others are used almost equally in two orders (e.g., *long and short* and *short and long*). In addition, binomials differ in idiomaticity (Moon, 1998). Some binomials are idiomatic (e.g., *sick and tired*), while the majority are literal, used by adding up the meaning of the individual elements (e.g., *men and women*). Moreover, the two content words of a binomial are often of close semantic relationships (Malkiel, 1959). Furthermore, studying first language (L1) - second language (L2) congruent binomials can help examine the congruency effect in MWE processing (Conklin and Carrol, 2019; Du et al., 2023; Chen, 2024). However, studies on MWE processing have mainly focused on collocations (e.g., Chen, 2024; Yi, 2018) and idioms (e.g., Carrol and Conklin, 2017, 2020; Jolsvai et al., 2020), while research on binomial processing, particularly L2 processing, is very limited (Mollin, 2014).

As such, the present study aims to examine Chinese EFL learners’ processing of English binomials and especially how it is influenced by the interlexical factors of L1-L2 congruency and L1 lexicalization and the intralexical factors of word and binomial frequency, binomial reversibility, and binomial predictability. It is hoped that the research results on binomial processing can not only deepen our understanding in its own right, but also shed new light on our understanding of the nature of MWE processing and the role of its influencing factors.

## 2 Literature review

As research on binomial processing is extremely limited (Mollin, 2014) and it has mostly been guided by the research literature on MWE processing (Siyanova-Chanturia et al., 2011), the following literature review will start with a discussion on the major factors influencing the L2 processing of MWEs and an elaboration on the main characteristics of English binomials, as they justify the selection of the influencing factors we focus on in analyzing binomial processing.

### 2.1 Major factors influencing the L2 processing of MWEs

#### 2.1.1 Frequency

Frequency is the most investigated factor influencing L2 MWE processing (Conklin and Thul, 2023), as research on frequency can shed light on the debate between usage-based theories of language acquisition (Bybee, 1998; Goldberg, 2006) and the words-and-rules approach (Pinker and Ullman, 2002). Studies have increasingly demonstrated that both L1 and L2 learning are sensitive to the frequency of all levels of linguistic units (Gries and Ellis, 2015), thus supporting the usage-based theories. For example, Wolter and Yamashita (2018) and Yi (2018) observed a phrasal frequency effect in both L1 and L2 speakers’ processing of English adjective + noun collocations and Chinese disyllabic adverbial collocations, respectively. Carrol and Conklin (2020) demonstrated that phrase frequency significantly contributes to the processing advantage of idioms, collocations, and binomials over their non-formulaic counterparts. Recently, Jiang and Siyanova-Chanturia (2023) showed that both L1 speakers and L2 learners are sensitive to phrase frequency manipulations in the processing of collocations.

Despite the “mounting evidence” demonstrating L1 and L2 learners’ sensitivity to the frequency of MWEs (Arnon and Snider, 2010, p. 67), researchers have increasingly recognized that frequency is just one of the factors influencing language acquisition and processing. Lexical factors and learners’ cognitive factors also play significant roles (Ellis, 2016). For example, Izura et al. (2011) found that age of acquisition (AoA) played a role in learning foreign words, independent of frequency factors. Jeong and Jiang (2019) revealed that both a high frequency of occurrence and structural completeness are necessary for the processing advantage of MWEs. As Ellis and Wulff (2020) noted, “frequency effects come in different kinds... and they will have differently weighted impacts depending on the target structure under examination” (p. 84). Therefore, studying the role of frequency in the processing of different MWEs is important for a better understanding of language learning. This is where the study of the role of frequency in binomial processing shows its value.

#### 2.1.2 L1-L2 congruency

Recent studies on the L2 processing of MWEs have increasingly focused on the influence of cross-language overlap and the L1-L2 congruency effect (Elgort et al., 2023), which postulates that L1-L2 congruent MWEs (i.e., L2 MWEs that have word-for-word translation equivalents in learners’ L1, Gyllstad and Wolter, 2016) are likely to be processed faster and/or more accurately than incongruent MWEs (Yamashita, 2018). This processing advantage has been reported for collocations (e.g., Yamashita and Jiang, 2010; Wolter and Yamashita, 2015, 2018) and idioms (e.g., Carrol and Conklin, 2014, 2017; Titone et al., 2015) (See Du et al., 2023, for a comprehensive review).

However, the underlying reasons of the congruency effect observed in MWE processing have remained unclear (Chen, 2024). A recent study by Yamashita (2018) on the possibility of semantic involvement in the congruency effect in collocations has offered new insights into this line of investigation. She re-categorized the congruent and incongruent collocations used in the studies of Wolter and Gyllstad (2011, 2013), Wolter and Yamashita (2015, 2018), and Yamashita and Jiang (2010) and found that most congruent collocations are transparent items and most incongruent collocations are opaque items, thus suggesting that semantic transparency may underlie the congruency effect. However, she cautioned against this quick conclusion and called for more studies on the semantic side of MWE processing. As the two constituent words of a binomial are of strong semantic relations, analyzing binomial processing may shed new light on our understanding of the congruency effect.

#### 2.1.3 Predictability

The predictability of MWEs refers to “the expectancy for the final word of a formulaic sequence, once the initial word or words have been seen” (Carrol and Conklin, 2020, p. 97). It has important implications for MWE processing, since more predictable MWEs are supposed to enjoy shorter processing times (Conklin and Thul, 2023).

However, only a handful of studies have investigated the role of predictability in MWE processing (e.g., Siyanova-Chanturia et al., 2011; Arcara et al., 2012; Carrol and Conklin, 2020) and there existed three different measures of predictability. One is the association strength between the two content words of an MWE. For instance, Siyanova-Chanturia et al. (2011) and Carrol and Conklin (2020) used the semantic association score from the Edinburgh Associative Thesaurus (EAT) database (Kiss et al., 1973) to index the predictability of an MWE. The second is transitional probability, which is calculated based on the whole phrase frequency and constituent word frequency (Frisson et al., 2005). For example, Carrol and Conklin (2020, p. 9) employed the formula “Overall Phrase Frequency ÷ Frequency of Word 1 × 100” to calculate the predictability of the second content word in a binomial given the first word. This method was also used in Arcara et al. (2012). The third measure is referred to as cloze probability, calculated by “the percentage of participants who provided the correct (or intended) completion” in a cloze test (Carrol and Conklin, 2020, p. 8).

As predictability is one of the typical features of binomials (Carrol and Conklin, 2020), the present study takes it as one focal point of investigation. Moreover, all three measures of predictability are employed to obtain a more comprehensive understanding of this feature and provide some reference for future studies.

### 2.2 Main characteristics of English binomials

The term “binomial” seemed to have made its first appearance in Malkiel’s (1959) seminal paper entitled *Studies in Irreversible Binomials*, defined as “the sequence of two words pertaining to the same form-class, placed on an identical level of syntactic hierarchy, and ordinarily connected by some kind of lexical link” (p. 113). Müller (2007) defined binomials in German as “complex idiomatic expressions consisting of two lexical items of the same category type that are connected by the conjunction *und* in German” (p. 29). More recently, Mollin (2014) defined binomials as “coordinated word pairs whose lexical elements share the same word class, such as *law and order*, *short and long*, *red and green*, or *rights and duties*” (p. 1), which is similar to Kopaczyk and Sauer’s (2017) definition presented in the Introduction part. It can be summarized from these four definitions that binomials typically consist of two words from the same word class, coordinated by a conjunction.

Besides these comparatively general definitions of binomials, studies operationalize binomials according to their particular research need. For example, in Carrol and Conklin (2020) binomials are defined as “combinations of *x-and-y* where a reversal of the order is entirely possible, but where one word order is highly conventionalized” (p. 4). Similar operational definition is used in Siyanova-Chanturia et al. (2011). In contrast, in Arcara et al. (2012), binomials are defined exclusively as being irreversible, and the order of the two constituents “cannot be reversed” (p. 1). It can be seen that the working definitions of binomials vary from study to study. Therefore, caution must be taken when making comparisons and generalizations across studies.

It should be noted that although there are cases of coordinated phrases such as *day in and day out*, multinomials with more than two lexical elements, such as *wine, women and song*, and binomials whose two elements are of different word classes, for example, *up and coming* (Norrick, 1988; Mollin, 2014), they are not dealt with in most of the studies on binomials. On reason is that they are not strictly covered by the above definitions of binomials. Another is that they are special cases which require separate consideration. Besides, binomials with two coordinated words that are not linked with the conjunction *and*, but with *or but* are also excluded in most of the studies. This is to concentrate on the most frequent and the most prototypical type of binomial, to ensure wide generalizations across studies (Mollin, 2014). This is also because binomials coordinated with the conjunctions *or* and *but* need to be studied separately. More importantly, in the context of the present study, only the A and B binomials are applicable to the analysis of L1-L2 congruency and L1-lexicalization, which are the key variables of the present study.

As such, the present study employs the definition by Mollin (2014), and a binomial is in the form of *A and B* in its preferred order, and *B and A* in its reversed and the less frequent form. Besides sharing some of the general features of other MWEs, binomials possess several unique characteristics, which need to be considered in analyzing its processing.

#### 2.2.1 Reversibility

Reversibility is the most distinctive feature of binomials. It was noted early by Abraham (1950) that some binomials are fixed in a definite order (e.g., *here and now*), and there are also many whose order can be reversed without changing its essential meaning (e.g., *ladies and gentlemen*). Malkiel (1959) further pointed out that binomials have different degrees of reversibility. For example, the frequency of *law and order* in the British National Corpus (BNC) is 587, while that of its reversed form *order and law* is 0; the frequencies of *up and down* and *down and up* are 2077 and 14; *long and short* and *short and long*, 61 and 60. Mollin (2014) proposed to measure binomial reversibility based on its corpus frequency by “freq ÷ (freq + revfreq) × 100” (p.40), in which “freq” represents the frequency of the more frequent, preferred binomial sequence, and “revfreq” is the frequency of the less frequent reversed sequence. Accordingly, the reversibility scores of the above three binomials are 100, 99.33, and 50.41, respectively. This score enjoys the simplicity of calculation and is intuitively understandable, allowing for statistical treatments of the reversibility of binomials. For example, Mollin (2014) investigated the similarities and differences between L1 and L2 speakers’ subjective judgment and the objective scores of the reversibility of 544 binomials. It was found that, L2 speakers’ judgment data in general shows a larger divergence from the English corpus data than the L1 speakers’, but more proficient learners do relatively better than less proficient learners, suggesting that they have had enough access to the language to build up a representation of the relative frequencies of binomial sequences, but not enough for the representation to be native-like. It lends support to the usage-based theories of language acquisition.

#### 2.2.2 Idiomaticity

Binomial idiomaticity is closely related to, but different from, its reversibility. According to Arcara et al. (2012), the reason why the order of an irreversible binomial cannot be reversed is that it has “an idiomatic, non-compositional meaning,” and “the reversed order is unacceptable” (p. 1), while the meaning of reversible binomials is referential, non-idiomatic and thus they can be freely reversed. Similarly, Kopaczyk and Sauer (2017) stated that irreversible binomials is irreversible since reversing their order would cancel the idiomatic meaning. In contrast, non-idiomatic binomials are fully transparent (Wolter, 2020) and are used “exactly to add up the meanings of the two individual elements” (Mollin, 2014, p. 37), and thus are reversible.

It can thus be inferred that relatively idiomatic binomials are generally irreversible, since their idiomaticity is the exact reason why they cannot be freely reversed, while reversible binomials are usually non-idiomatic and semantically transparent and can be reversed without altering the basic meaning. Therefore, idiomaticity and semantic transparency mean the same for binomials. However, they are different from binomial reversibility. Mollin (2014) analyzed and compared the (ir)reversibility and idiomaticity of 544 frequent binomials in the BNC and all the idiomatic binomials in three English idiom dictionaries. The data revealed that irreversibility often co-occurs with idiomaticity: idiomatic binomials are mostly irreversible and non-idiomatic ones are often reversible. However, the data also showed that a large number of irreversible binomials are not idiomatic and a small number of idiomatic binomials are reversible, and she concluded that irreversibility and idiomaticity are not equivalent. Therefore, binomial irreversibility and idiomaticity should be treated and analyzed separately. Moreover, in Mollin’s study (2014) of L1 and L2 speakers’ subjective judgment of the reversibility of binomials, it was found that idiomatic or opaque binomials cause more judgment problems for L2 speakers than L1 speakers, which calls for investigation into L2 speakers’ treatment of idiomatic or opaque binomials in more perspectives.

### 2.3 Binomial processing

Psycholinguistic studies on binomials have been “extremely scarce” (Mollin, 2014, p. 5). The number of studies on binomial processing is very limited. To the best of the author’s knowledge, there were only five such studies to date.

To begin with, Siyanova-Chanturia et al. (2011) conducted an eye-tracking study on 30 reversible binomials and their reversed forms to explore the role of phrasal frequency in L1 and L2 speakers’ processing of MWEs. Mixed-effects modeling revealed that both L1 and L2 speakers were sensitive to whole phrase frequency. Results also show that L1 speakers and higher proficiency L2 speakers but not lower proficiency L2 speakers were sensitive to phrase type (binomial vs. reversed). This suggested that higher proficiency L2 speakers developed mental representations of the phrase, similar to L1 speakers, while lower proficiency subjects have not encountered the items sufficiently enough to form entrenchment in memory. The result was taken to demonstrate that both L1 and L2 speakers’ language processing is subject to the frequency and later entrenchment of all levels of input, supporting usage-based models of language processing. In spite of the insights their study provide, there are two points which need special attention. First, processing advantage was found for both phrase frequency and phrase type (preferred order vs. reversed order) and phrase frequency effect has remained when phrase type effect was singled out. Second, it is specified in the material selection criterion that only highly frequent binomials were used as the target material and that the frequency of the stimulus differed significantly from the frequency of their reversed order counterparts (e.g., 247.3 vs. 27.4 occurrences per 100 million words in the BNC). These two facts combined together mean only highly frequent and less reversible binomials were considered in their study, and the research finding might not as valid as when binomials of all frequency bands and all reversibility bands were considered. The present study takes this limitation into consideration and attempts to cover a wide range of binomials.

Moreover, as Siyanova-Chanturia et al. (2011) themselves pointed out that the phrase type effect found for binomials could not be taken to mean holistic processing of the preferred order binomials, since binomials are characterized by having strong semantic associations between the two constituent word, which is referred to as having high “predictability” (p. 782). To partial out the possible effect of predictability from the phrase type effect, they used cloze probability (i.e., number of subjects who correctly complete the structure *A and ___*, and *B and ____*) as a measure of predictability and found that phrase type effect still remained when cloze probability scores were regressed out. However, besides the potential bias in selecting the stimuli mentioned above, whether cloze probability is a reliable measure of predictability remains unclear.

Regarding these potential limits of their study, the present study attempts to address these issues by investigating a more representative range of binomials and employing all three existing indexes to measure binomial predictability.

Quite different from Siyanova-Chanturia et al. (2011), Arcara et al. (2012) explored L1 speakers’ representation and processing of irreversible binomials (IBs), which were idiomatic with a relatively fixed word order. Therefore, a change of word order would result in a complete change of meaning (e.g., *hit and run*). Uniquely, the subjects in the study were patients of neglect dyslexia, who are characterized by having problems reading letters, words, or other sentence fragments located on the left visual space. They were asked to read aloud 36 IBs, their reversed forms (RBs), and 36 non-binomials. The underlying rationale was that if IBs enjoyed a lexical effect, that is, if they were stored and processed as a whole, then these patients would read IBs more accurately than RBs and non-binomials. The results did show that the patients read IBs in their correct order with fewer errors, and thus the researchers concluded that IBs were stored in the mental lexicon as a whole and were retrieved as such. One point open to discussion in their study is that, as in Siyanova-Chanturia et al. (2011), Arcara et al. (2012) also claimed that the only difference between the stimuli and their reversed forms was “the order of constituents” (p. 7). However, it may be argued that IBs and RBs differed in their status as a formulaic phrase, and more importantly, IBs had meaning, while RBs were mostly intelligible, and IBs were far more frequent than RBs in the language input. The processing advantage of IBs may be because they were semantically intelligible and more frequent than RBs and thus led to faster activation, but unlike Siyanova-Chanturia et al. (2011), Arcara et al. (2012) did not find whole-word frequency effect within the IBs and they did not check the whole word frequency effect across all stimuli. Therefore, it is still debatable whether a lack of frequency effect within IBs in their study could be used as evidence against a frequency effect across all the stimuli. Besides, as Siyanova-Chanturia (2015) argued, faster processing of formulaic language over its novel counterpart could not be taken to mean holistic storage and processing.

In all, Arcara et al.’s (2012) study on Italian neglect dyslexia patients’ processing of IBs bears important implications for the representation and processing of binomials, although the implications are limited by the specific type of stimuli and the subjects employed in the study.

To further test the possible role of predictability in binomial processing, Siyanova-Chanturia et al. (2017) investigated L1 speakers’ electrophysiological responses to highly frequent, familiar, and predictable binomials in terms of event-related potentials (ERPs). Using the same stimuli in Siyanova-Chanturia et al. (2011), they found that compared with infrequent binomials and semantically infelicitous two-word phrases (i.e., binomials without the conjunction *and*, such as *knife-fork*), the target stimuli elicited larger P300s and smaller N400s, which indicates less processing load and smoother semantic integration, possibly resulted from the activation of a mental “template” of binomials (i.e., *___ and ___*). When the template is taken away in their Experiment 2, that is, when the *A and B* structure of binomials was replaced with *A B*, no difference in P300s and N400s was observed for the three types of stimulus. This further confirms the important role of the binomial configuration entrenched in the brain and lends support to the usage-based and exemplary-based models of language processing, as Siyanova-Chanturia et al. (2011) did. Particularly, their study adds to the increasing evidence of the frequency-based accounts of language acquisition from a different perspective through the ERP method. It also draws attention to the study of the role of predictability in MWE processing in future studies.

More recently, Carrol and Conklin (2020) compared L1 English speakers’ processing of idioms, binomials, and collocations through eyetracking. The results showed a processing advantage for all three types of MWEs over their controls and frequency was its main drive. Moreover, they found that different types of MWEs were influenced by their specific distributional properties: idioms by familiarity and decomposability, binomials by predictability and semantic association, and collocations by mutual information. The study is enlightening in that it took a relatively integrated view of the subtypes of formulaic language, considering their commonalities as well as specificities and in doing so, they were able to arrive at more informed conclusions. It also demonstrates that different subtypes of MWEs should be studied with a focus on their unique features. Besides, their finding concerning the significant role of predictability in binomial processing justifies our interest in this particular feature of binomials.

Du et al. (2021) is by far the only study investigating the cross-language influence in binomial processing. Two groups of bilinguals (Chinese-English and English-Chinese) and a control group of English monolinguals performed a visual lexical decision task with three types of binomials: congruent, English-only, and Chinese-only (Chinese binomials translated into English), and their matched controls. It was found that Chinese-English bilinguals showed a significant priming effect for congruent binomials but not for English-only binomials, English-Chinese bilinguals showed no significant priming for either congruent or English-only binomials, and English monolinguals showed comparable priming for both congruent and English-only binomials. Moreover, none of the groups showed priming for translated Chinese-only binomials over their controls. The authors concluded that there are L1 influences in L2 binomial processing and possibly a lesser degree of cross-linguistic influences from the L2 to L1.

The studies on binomial processing, though limited in number, have helped us understand more of the role of predictability and congruency in MWE processing, but their varied nature makes it difficult for cross-study comparisons, for example, the binomials used in the studies cannot be treated as of the same nature. Specifically, in Arcara et al. (2012) all target items were idiomatic as well as irreversible, while in Carrol and Conklin (2020) all were non-idiomatic and entirely reversible. Therefore, more studies are needed in this line of research. Such studies are especially valuable considering the research advantage of binomials compared with other types of MWEs (see Siyanova-Chanturia et al., 2011 and Mollin, 2014, for more detailed discussions).

### 2.4 English binomials vs. Chinese binomials

The use of binomials is a common linguistic phenomenon across different languages and there are also a large number of binomials in Chinese (Wang and Gu, 1988). Many English binomials can be translated literally word-for-word into Chinese binomials, as being L1-L2 congruent. There are also binomials, particularly those with idiomatic meanings, which cannot be translated word-for-word into Chinese. To convey the same meaning, different lexical items or paraphrasing is needed in Chinese.

Many Chinese binomials are further lexicalized into two-character coordinated compounds (Zhang, 2000), omitting the conjunction “*and*” (represented as “*he*” in Chinese *pinyin*) (Xing, 1991). Consequently, among English-Chinese congruent binomials, some can be translated not only into Chinese binomials, but more appropriately and naturally into coordinated compounds. In other words, they can be translated into words that are part of the established vocabulary or are “lexicalized” (Flyxe, 2002). Zhang (2000) and other comparative studies have discussed this phenomenon extensively and provided many examples. For instance, when “*men and women*” is translated word-for-word into Chinese, it becomes “*nan he nv*,” with “*nan*” for *men*, “*nv*” for *women*, and “*he*” for the conjunction *and*. However, Chinese word pairs like “*nan he nv*” are often used as lexicalized coordinated compounds like “*nan nv*,” without the conjunction “*he*.”

Paribakht (2005) demonstrates that Farsi EFL learners struggle to decode the meaning of nonlexicalized words when reading English texts containing both lexicalized and nonlexicalized words, which suggests that L1 lexicalization is a potential factor influencing learners’ L2 reading and lexical development. Extending this possibility in the context of L2 binomials, it is worth exploring whether L1 lexicalization, in addition to congruency, affects Chinese EFL learners’ processing of English binomials.

## 3 Research questions

As such, the study addresses the following research questions (RQs).

RQ1: What are the descriptive characteristics of Chinese EFL learners’ processing (accuracy and reading times) of English binomials?

RQ2: How is their binomial processing (accuracy and reading times) affected by the interlexical factors of L1-L2 congruency and L1 lexicalization?

RQ3: How is their binomial processing (accuracy and reading times) affected by the intralexical factors of word and binomial frequency, binomial reversibility, and binomial predictability?

## 4 Methodology

### 4.1 Participants

Forty Chinese postgraduate EFL learners from a university in China took part in the main study on a voluntary basis and the study was conducted with the ethics approval of their university. They were administered a background questionnaire and the updated VLT at the 3,000 word level (Webb et al., 2017). The data is listed in Table 1. The subjects scored a mean of 81.875 (*SD* = 6.374) out of the full score of 90 on the 3,000 word level VLT, suggesting an adequate mastery (Schmitt et al., 2001).

**Table 1:** Biographical data of the participants (*N* = 40; male/female = 30/10).

Variable	Mean	SD	Min.	Max.
Age	26.05	4.29	22	39
AOS	10.975	2.626	6	18
LOR	0.125	0.463	0	2
VLT-3000	81.875	6.374	67	90

AOS, age of starting English learning; LOR, length of residence in English speaking countries.

### 4.2 Materials

#### 4.2.1 Critical stimuli

The critical stimuli were 64 English binomials. The key categorical variables were L1-L2 congruency and L1 lexicalization. As discussed in the review, congruency is judged depending on the existence of a word-for-word translation equivalent in the L1. Congruent binomials are further divided into L1-lexicalized and L1-nonlexicalized binomials, depending on whether they can be translated into Chinese two-character coordinated compounds. Their data sources included (1) Mollin’s (2014) list of 544 binomials chosen from the BNC; (2) more than 200 idiomatic binomials retrieved from three English idiom dictionaries (Sinclair and Moon, 1995; McCarthy and Walter, 1998; Spears, 2005); (3) the binomials analyzed in some Chinese studies on English binomial (e.g., Wang, 1986; Wang and Gu, 1988; Wang, 2000; Zhang, 2000; Li and Wei, 2012).

The preliminary selection of the binomials was done by the researcher, through which proper nouns, those having clear cultural connotations and those whose constituent words show significant difference in length and structure were excluded. It was also made sure that all of the constituent words of the binomials are in Nation’s first 3,000 BNC/COCA headword list (Nation, 2017), to guarantee that the constituent words were known to the subjects, because unfamiliarity with the stimuli would reduce the reliability of the results (Sonbul and El-Dakhs, 2020).

From the resultant set of binomials, the researcher selected the ones that are normally translated into two-character Chinese coordinated compounds, which resulted in a further list of 58 binomials. This list was then used in a norming test to generate the target stimuli of L1-lexicalized binomials. The rest binomials in the set were used to generate the target stimuli of L1-nonlexicalized binomials. In the norming test, 25 EFL learners from the same population with the main subjects were asked to translate the 58 binomials into two-character Chinese compounds. 16 of the binomials that were translated by at least 23 subjects with the same answers were used as the L1-lexicalized binomial stimuli. The researcher then chose 16 L1-nonlexicalized binomials matched with them in word and binomial frequency as well as length. The 32 incongruent binomials were selected solely from the more than 200 binomials retrieved from the three idiom dictionaries mentioned above and matched to the congruent items in word and binomial frequency, as well as word and binomial length.

The constituent words of the final 64 stimuli all belong to Nation’s first 3,000 headword list (2017). In fact, except for *ache*, *phrase*, and *theory*, all other words are from Nation’s first 2000 headword list. The 64 critical stimuli are listed in Table A1.

#### 4.2.2 Descriptive statistics of the critical stimuli

Values of frequency (i.e., word and binomial frequency) are retrieved from the BNC. Binomial reversibility is calculated with Mollin’s (2014) formula presented earlier. For the three measures of binomial predictability, association strength is obtained from the EAT (Kiss et al., 1973) and the Small World of Words (SWOW) word association norms (De Deyne et al., 2018); transitional probability is computed with Carrol and Conklin’s (2020) formula presented in the review; values of cloze probability were obtained from a binomial completion test of the target binomials with a matched group of 214 students from the same population as the main subjects. In the test, they were instructed to provide a word that they think is the most appropriate to fill into the blanks in the binomial phrases. The cloze probability score of a certain binomial was calculated as the percentage of participants who provided the correct words. Table 2 summarizes the descriptive statistics of the critical stimuli and the *t*-tests of different types of items.

**Table 2:** Summary of item properties and *t*-tests of different types of items.

	Pair		t	p	Pair		t	p
	Congruent (*n* = 32) M (SD)	Incongruent (*n* = 32) M (SD)			Congruent L1-lexicalized (*n* = 16) M (SD)	Congruent L1-nonlexicalized (*n* = 16) M (SD)		
W1 frequency (log)	2.35 (0.43)	2.32 (0.94)	0.128	0.899	2.45 (0.38)	2.24 (0.47)	1.438	0.161
W2 frequency (log)	2.11 (0.38)	2.26 (0.81)	−0.905	0.370	2.23 (0.34)	2.00 (0.39)	1.730	0.094
Binomial frequency (log)	2.32 (0.35)	2.20 (0.42)	1.204	0.233	2.25 (0.38)	2.38 (0.32)	−1.026	0.313
W1 length	4.66 (1.56)	4.06 (0.98)	1.824	0.074	4.25 (0.93)	5.06 (1.95)	−1.505	0.147
W2 length	5.00 (1.44)	4.59(1.13)	1.256	0.214	4.63 (1.02)	5.38 (1.71)	−1.506	0.145
Binomial length	9.66 (2.70)	8.66 (1.81)	1.743	0.087	8.88 (1.63)	10.44 (3.33)	−1.688	0.106
Reversibility	86.05(13.68)	97.00(5.08)	−4.245	0.000	86.78 (13.95)	85.33 (13.82)	0.295	0.770
EAT	0.57 (0.51)	0.54 (0.44)	0.278	0.782	0.79 (0.43)	0.35 (0.49)	2.636	0.013
SWOW	0.17 (0.13)	0.19 (0.15)	−0.516	0.607	0.24 (0.12)	0.11 (0.09)	3.415	0.002
Transitional probability	0.04 (0.04)	0.08 (0.15)	−1.389	0.173	0.03 (0.02)	0.06 (0.05)	−2.749	0.011
Cloze probability	0.58 (0.25)	0.49 (0.25)	1.524	0.132	0.69 (0.16)	0.47 (0.28)	2.837	0.008

log: log transformed.

It can be seen from Table 2 that congruent and incongruent items are matched in all variables except for reversibility. This is natural and unavoidable considering that incongruent items are idiomatic and are hardly used in their reversed forms (Mollin, 2014). Congruent L1-lexicalized and L1-nonlexicalized binomials are matched in all but predictability measures. This data is interesting as it seems that binomials with a closer semantic or lexical relationship are lexicalized in Chinese.

#### 4.2.3 Baseline items

Sixty-four baseline items were constructed by recombining the constituent words of the critical stimuli to avoid differences in constituent word frequency between the target and the baseline items, a method proposed by Wolter and Yamashita (2015). It was also ensured that the two content words of a baseline item are of the same word class, following the definition of binomials, and the resultant combinations are nonexistent in the BNC. The baseline items are listed in Table A2.

### 4.3 Procedure

A phrase acceptability judgment task was administered following the literature (e.g., Wolter and Gyllstad, 2013; Wolter and Yamashita, 2018), as it taps into participants’ processing of meaning (Chen, 2024) and thus better suits the purpose of the present study. The task was programmed and administered using E-prime software (Schneider et al., 2002) running under Windows XP OS on a standard desktop PC.

The test started with a practice session with 15 practice items. The main session with the 128 target items followed once the subjects were familiar with the task instruction. All items were presented to the subjects in a personalized randomized order. Each trial ran in the following procedure. First, a fixation target of 12 red asterisks was presented in the middle of the screen for 250 ms. Then a blank screen was shown for 66 milliseconds. It was followed by the presentation of a target item, which was presented in red in 18-point Courier New font in the center of the screen on a white background. The presentation of the item ended once the subject made a response or it timed out at 4000 milliseconds. For each item, the subjects were asked to judge whether “the word combinations were commonly used in English” (Wolter and Yamashita, 2018, p. 402) and they were instructed to respond quickly by pressing the “J” key for “Yes,” and the “F” key for “No.” The subjects’ reaction time in milliseconds and “yes/no” responses were logged.

The whole procedure was piloted with 5 students from the same population as the subjects before the formal data collection. No administration issues were revealed.

### 4.4 Analysis

The independent variables include two categorical variables of L1-L2 congruency and L1 lexicalization, and numerical variables of constituent word frequency, binomial frequency, reversibility scores, and predictability values in terms of EAT, SWOW, transitional probability and cloze probability.

The data analysis was carried out using R version 4.2.1 (R Core Team, 2022). Before data analysis, several data cleaning steps were applied to the 5,120 responses from the 40 participants. First, the data of four participants whose error rates were higher than 30% were excluded. Second, the response time of erroneous responses was excluded, resulting in a data loss of 19.03%. Third, as in Wolter and Yamashita (2018), the responses with a response latency less than 450 milliseconds were excluded, which accounted for 0.05% of the data. In all, the data trimming procedure excluded 27.17% of the data. The response time of the remaining data was then log-transformed to reduce skewness in the distribution. All other continuous variables were centered and standardized. Before model fitting, the assumptions of multiple regressions (Levshina, 2015) were checked and no issues were revealed. The Variance Information Factor (VIF) values were calculated using the *car* package in R (Fox and Weisberg, 2019), which range from 1.118 to 3.223, revealing no collinearity among the predictor variables. The data analyses were then conducted with mixed-effects modeling using the *lme4* package (Bates et al., 2015) in R.

Eight simple models which included the baseline items (four for response time and four for accuracy) were constructed to answer RQ2; two complex models which excluded the baseline items (one for response time and one for accuracy) were constructed to investigate RQ3. Similar approaches are employed by Wolter and Gyllstad (2013) and Öksüz et al. (2021). Furthermore, the effects of type and condition were analyzed in separate models. All models included random intercepts for subjects and items and fixed effects of the interested variables.

The modeling procedure ran in the following steps. First, the predictor of stimulus type was coded so that the following four sets of comparisons were made: (1) between baselines and binomials; (2) between incongruent and congruent binomials; (3) between congruent L1-lexicalized and L1-nonlexicalized binomials; (4) between incongruent binomials and the other three types (Reference level: Incongruent). The first three comparisons were made based on contrast-coding of the predictor and the last comparison on dummy coding. The simple models included fixed effects of stimulus type, self-rated proficiency, and VLT. For the complex models, a maximal model was built with all frequency, reversibility, and predictability variables as the fixed effects. In total, ten models were constructed.

## 5 Results

### 5.1 Results of RQ1

RQ1 investigates the descriptive characteristics of Chinese EFL learners’ processing (reading time and accuracy) of English binomials. Table 3 presents the means and *SD*s of log transformed response time and accuracy. Item type and condition are distinguished. Type refers to three item types, including congruent binomials, incongruent binomials, and non-binomial baselines; condition refers to four item conditions, including congruent L1-lexicalized binomials, congruent L1-nonlexicalized binomials, incongruent binomials, and non-binomial baselines.

**Table 3:** Means (*SD*s) of log transformed response time (logRT) and accuracy by type and condition.

Type	logRT	Accuracy	Condition	logRT	Accuracy
Congruent	7.24 (0.34)	0.86 (0.34)	Congruent L1-lexicalized	7.21 (0.33)	0.9 (0.3)
			Congruent L1-nonlexicalized	7.27 (0.34)	0.82 (0.38)
Incongruent	7.25 (0.38)	0.69 (0.46)	Incongruent	7.25 (0.38)	0.69 (0.46)
Baseline	7.41 (0.33)	0.79 (0.4)	Baseline	7.41 (0.33)	0.79 (0.4)

It shows from Table 3 that compared with incongruent binomials (*M* = 7.25, *SD* = 0.38 and *M* = 0.69, *SD* = 0.46) and baseline items (*M* = 7.41, *SD* = 0.33 and *M* = 0.79, *SD* = 0.4), congruent binomials obtained the lowest mean response time (*M* = 7.24, *SD* = 0.34) and highest accuracy (*M* = 0.86, *SD* = 0.34). Compared with baseline items, incongruent binomials on average got shorter reaction time but lower accuracy. Among congruent binomials, congruent L1-lexicalized binomials obtained lower mean response time (*M* = 7.21, *SD* = 0.33) and higher accuracy than L1-nonlexicalized binomials (*M* = 7.27, *SD* = 0.34 and *M* = 0.82, *SD* = 0.38).

### 5.2 Results of RQ2

The results of the eight simple models constructed to answer RQ2 on the influence of L1-L2 congruency and L1 lexicalization on the subjects’ processing time and accuracy are presented in the following.

#### 5.2.1 Effects of being binomials: binomials VS baseline items

The mean response time for binomials is significantly shorter than that for the baselines (*β* = −0.06, *t* = −5.07, *p* = <0.001), but they show no significant difference in accuracy (*β* = 1.03, *z* = 0.51, *p* = 0.610) (See Table 4).

**Table 4:** Results of the simple models comparing binomials and baselines.

Models	Predictors	Estimates	std. error	Statistic	p
Response time	(Intercept)	7.32	0.02	305.40	<0.001
	Binomial items	−0.06	0.01	−5.07	<0.001
Accuracy	(Intercept)	5.29	0.14	11.84	<0.001
	Binomial items	1.03	0.05	0.51	0.610

#### 5.2.2 Effects of L1-L2 congruency: congruent binomials VS incongruent binomials

Table 5 shows that there is no significant difference between incongruent and congruent binomials in response time (*β* = −0.00, *t* = −0.18, *p* = 0.861), while the mean accuracy of incongruent binomials is significantly lower than that of congruent binomials (*β* = 2.06, *z* = 4.41, *p* < 0.001).

**Table 5:** Results of the simple models comparing incongruent and congruent binomials.

Models	Predictors	Estimates	std. error	Statistic	p
Response time	(Intercept)	7.35	0.02	306.93	<0.001
	Congruency	−0.00	0.03	−0.18	0.861
Accuracy	(Intercept)	5.60	0.13	13.55	<0.001
	Congruency	2.06	0.16	4.41	<0.001

#### 5.2.3 Effects of L1 lexicalization: L1-lexicalized VS L1-nonlexicalized binomials

There is a significant difference between L1-lexicalized and L1-nonlexicalized binomials in accuracy (*β* = 0.53, *z* = −1.61, *p* = 0.018) but not in response time (*β* = 0.07, *t* = 1.19, *p* = 0.235) (See Table 6).

**Table 6:** Results of the simple models comparing congruent L1-lexicalized and L1-nonlexicalized binomials.

Models	Predictors	Estimates	Std. error	Statistic	p
Response time	(Intercept)	7.35	0.02	309.98	<0.001
	L1-lexicalization	0.07	0.06	1.19	0.235
Accuracy	(Intercept)	5.16	0.13	12.61	<0.001
	L1-lexicalization	0.53	0.40	−1.61	0.018

#### 5.2.4 Effects of condition: comparing four item conditions

To compare four item conditions, the predictor of condition was dummy coded so that incongruent binomials were taken as the reference level. As shown in Table 7, no significant difference was observed in response time either between incongruent binomials and congruent L1-lexicalized binomials (*β* = −0.08, *t* = −1.79, *p* = 0.075) or between incongruent and congruent L1-nonlexicalized binomials (*β* = −0.01, *t* = −0.32, *p* = 0.751). However, both congruent L1-lexicalized and L1-nonlexicalized binomials were processed with significantly higher accuracy than incongruent binomials (Incongruent vs. Congruent L1-lexicalized: *β* = 0.21, *z* = 4.82, *p* < 0.001; Incongruent vs. Congruent L1-nonlexicalized: *β* = 0.14, *z* = 3.11, *p* = 0.002). In addition, incongruent binomials were processed with significantly faster speed but lower accuracy than baseline items (Response time: *β* = 0.10, *t* = 3.35, *p* = 0.001; Accuracy: *β* = 0.10, *z* = 3.39, *p* = 0.001).

**Table 7:** Results of the simple models comparing four item conditions.

Models	Predictors	Estimates	std. error	Statistic	p
Response time	(Intercept)	7.31	0.03	227.35	<0.001
	Baseline	0.10	0.03	3.35	0.001
	Congruent L1-lexicalized	−0.08	0.04	−1.79	0.075
	Congruent L1-nonlexicalized	−0.01	0.04	−0.32	0.751
Accuracy	(Intercept)	0.69	0.03	24.28	<0.001
	Baseline	0.10	0.03	3.39	0.001
	Congruent L1-lexicalized	0.21	0.04	4.82	<0.001
	Congruent L1-nonlexicalized	0.14	0.04	3.11	0.002

### 5.3 Results of RQ3

Table 8 presents the results of the two complex models which excluded the data of the baseline items to answer RQ3 about the effect of intralexical factors of word and binomial frequency, binomial reversibility, and binomial predictability on the subjects’ processing time and accuracy. As it shows, there is no significant effects of frequency variables on response time or accuracy in terms of (1) Word 1 frequency (Response time: *β* = 0.01, *t* = 0.31, *p* = 0.760; Accuracy: *β* = 1.03, *z* = 0.13, *p* = 0.900), (2) Word 2 frequency (Response time: *β* = −0.07, *t* = −1.72, *p* = 0.090; Accuracy: *β* = 1.45, *z* = 1.24, *p* = 0.215), and (3) binomial frequency (Response time: *β* = −0.06, *t* = −1.07, *p* = 0.289; Accuracy: *β* = 1.75, *z* = 1.36, *p* = 0.175). Binomial reversibility scores significantly predicted learners’ judgment accuracy (*β* = 0.98, *z* = −2.15, *p* = 0.032), but not response time (*β* = 0.00, *t* = 0.72, *p* = 0.473), which means that the more reversible the binomial, the less accurate its processing. Binomial predictability measured with cloze probability significantly affected learners’ processing time and accuracy (*β* = −0.35, *t* = −3.52, *p* = 0.001 and *β* = 41.90, *z* = 4.79, *p* < 0.001, respectively), which shows that the higher the cloze probability, the shorter the processing time and the higher the processing accuracy; the other indices of binomial predictability did not predict response time or accuracy: (1) EAT (Response time: *β* = −0.08, *t* = −1.36, *p* = 0.181; Accuracy: *β* = 1.11, *z* = 0.24, *p* = 0.812); (2) SWOW (Response time: *β* = 0.04, *t* = 0.30, *p* = 0.765; Accuracy: *β* = 0.43, *z* = −0.76, *p* = 0.447); and (3) Transitional probability (Response time: *β* = −0.02, *t* = −0.11, *p* = 0.912; Accuracy: *β* = 0.54, *z* = −0.38, *p* = 0.705).

**Table 8:** Results of the complex models for response time and accuracy.

	Response time				Accuracy			
Predictors	Estimates	Std. error	Statistic	p	Odds ratios	Std. error	Statistic	p
(Intercept)	7.29	0.02	344.67	<0.001	6.06	0.17	10.60	<0.001
Word 1 frequency	0.01	0.03	0.31	0.760	1.03	0.23	0.13	0.900
Word 2 frequency	−0.07	0.04	−1.72	0.090	1.45	0.30	1.24	0.215
Binomial frequency	−0.06	0.05	−1.07	0.289	1.75	0.41	1.36	0.175
Reversibility score	0.00	0.00	0.72	0.473	0.98	0.01	−2.15	0.032
EAT	−0.08	0.06	−1.36	0.181	1.11	0.44	0.24	0.812
SWOW	0.04	0.15	0.30	0.765	0.43	1.09	−0.76	0.447
Transitional probability	−0.02	0.21	−0.11	0.912	0.54	1.61	−0.38	0.705
Cloze probability	−0.35	0.10	−3.52	0.001	41.90	0.78	4.79	<0.001

## 6 Discussion

### 6.1 Characteristics of Chinese EFL learners’ binomial processing

Several noteworthy features emerge from analyzing Chinese EFL learners’ response time and accuracy data in the binomial judgment task.

First, the learners exhibited difficulty judging whether an *A and B* phrase is binomial or not. This is inferred from facts that 19.03% of the judgment data consisted of erroneous responses, but the subjects were familiar the constituent words of the test items. This finding is consistent with the result reported by Boonnoon (2020) that in his study, 130 Thai university students obtained an average of 17.91 out of a total score of 40 in an offline binomial acceptability judgment test, indicating a low level of knowledge of English binomials. The finding also aligns with previous research demonstrating that L2 learners often fail to recognize MWEs as regularly occurring, routinized, and formulaic expressions (Wray, 2002; Wolter, 2020). For example, Bishop (2004) found that even with appropriate exposure, learners may fail to recognize the formulaic sequences in the language input. Laufer and Waldman (2011) also argued that language learners may not recognize MWEs that are relatively semantically transparent and composed of highly frequent words as prefabricated, but as free combinations of two words or literal translations of their L1 equivalents.

The present study contributes to the literature by revealing that binomials also pose challenges for L2 learners like other types of MWEs, such as collocations (Boers, 2020) and idioms (Siyanova-Chanturia and Lin, 2018). Their pervasive structure may render them even more elusive.

Secondly, the subjects processed both congruent and incongruent binomials significantly faster than baseline items. This finding aligns with previous studies showing that L2 speakers process MWEs faster than matched non-formulaic controls (Siyanova-Chanturia et al., 2011; Hernández et al., 2016).

However, it was found that while congruent binomials were processed significantly more accurately than baseline items, incongruent binomials obtained lower accuracy than baseline items. The latter finding may seem counter-intuitive, but it can be explained by the fact that the incongruent binomials were all idiomatic, such as *forgive and forget* and *far and wide*, and previous literature has demonstrated L2 speakers’ difficulty in processing idiomatic sequences in the L2. For example, in the eye-tracking experiment by Carrol and Conklin (2017), Chinese L1 speakers showed no difference between English idioms and their literally plausible but nonexistent controls, and they read the literal versions of idioms faster than their figurative equivalents. Moreover, research has demonstrated that learners tend to interpret the figurative meaning of an idiom through a literal reading of the expression (Cieślicka, 2015). In the present study, literal interpretations of incongruent binomial stimuli are semantically possible. In contrast, the baseline items are not semantically sensible (e.g., *alive and small* and *doctors and phrases*), so once the subjects recognized the constituent words of a baseline, they would be able to make an accurate judgment. This also suggests that the subjects had knowledge of the binomial construction and its possible internal semantic relationships (Mollin, 2014).

### 6.2 Effects of inter-lexical factors on binomial processing

L1-L2 congruency was found to have a significant effect on the learners’ processing accuracy but not on reaction time. This processing advantage of congruent binomials over incongruent binomials echoes the congruency effect reported on collocations (e.g., Wolter and Yamashita, 2018; Chen, 2024) and idioms (e.g., Carrol and Conklin, 2014, 2017; Titone et al., 2015). It adds to the finding of Du et al. (2021) that the Chinese-English bilinguals showed a significant priming effect for congruent binomials over their corresponding controls in a lexical decision task.

Regarding L1-lexicalization, it is revealed that L1-lexicalized binomials were processed significantly more accurately than nonlexicalized ones. Titone et al. (2015) proposed distinguishing between formal, semantic or conceptual, and pragmatic levels of congruency, since different types and even degrees of congruency are likely to influence language learning differently. The congruent L1-nonlexicalized binomials in the present study can be classified as having semantic congruency, while congruent L1-lexicalized binomials show both formal and semantic congruency. Therefore, the present finding of the significant role of L1-lexicalization provides empirical evidence for the argument of degrees and types of L1-L2 congruency effect. As such, this study proposes differentiating and analyzing different types and levels of L1-L2 congruency in MWE processing, to help shed light on the underlying mechanisms of the congruency effect.

### 6.3 Effects of intralexical factors on binomial processing

First, there is no significant effects of word or binomial frequency on the speed or accuracy of L2 binomial processing. This result expands the finding of Arcara et al. (2012) that no frequency effect was found in the L1 processing of irreversible binomials and is consistent with the finding of Du et al. (2021) that the Chinese-English bilinguals showed no significant main effects of phrase frequency in L2 binomial processing.

However, this seems to run counter to the word and/or phrasal frequency effects widely reported for other types of MWEs (e.g., Hernández et al., 2016; Chen, 2024). Nonetheless, in the present study, the constituent words of the stimuli are highly frequent, which may have created a ceiling effect. As for binomial frequency, its role is likely to be mediated or offset by learners’ sensitivity to the close semantic and/or formal relationship between the two constituent words. Take the target binomials *long and short*, *good and bad*, and *men and women* as examples: their frequency in the BNC is 61, 194, and 1949, respectively. Although the three binomials differ significantly in frequency, their constituent words all show a clear and close semantic relationship (Malkiel, 1959), making it easy for learners to judge that the three combinations are commonly used in English.

A recent study by Jolsvai et al. (2020) also points to the important role of the semantic aspect in MWE processing, independent of frequency. They found that sequence meaningfulness directly affected learners’ processing speed, independent of chunk frequency in the processing of three-word sequences. In addition, the *A and B* template or configuration has been shown to be crucial for the processing advantage of binomials, without which the processing advantage observed for binomials over infrequent strongly associated phrases and semantic violations no longer existed (Siyanova-Chanturia et al., 2017). Moreover, the semantic relationship between the constituent words and the unique formal configuration are two primary features of binomials (Malkiel, 1959). As such, future studies on binomial processing should take these features into consideration. Nonetheless, the present study showed that L2 learners’ knowledge of the semantic and formal features of binomials played a role in their binomial processing, independent of frequency.

Secondly, binomial reversibility was found to have significant negative effects on learners’ processing accuracy, that is, less reversible binomials (i.e., mostly used in only one order) obtained lower processing accuracy. It mirrors the finding that congruent binomials were processed with significantly higher accuracy than incongruent ones, since incongruent binomials (all idiomatic) are significantly less reversible than congruent binomials (all literal) as shown in Table 2. Though this poses questions about the overlap of variables, it is impossible to separate idiomaticity from congruency, at least for L2 English binomials with Chinese as L1. Similar cases exist in the literature for other MWEs with different L1s. In Yamashita and Jiang (2010), many incongruent collocations between English and Japanese are idiomatic, (e.g., *kill time*), while most congruent items (e.g., *heavy stone*) are completely literal. Similarly, in Wolter and Gyllstad (2011, 2013), most congruent collocations between English and Swedish are completely literal (e.g., *give an answer*), while most incongruent items are at least partially figurative (e.g., *pay a visit*), because otherwise, a literal translation between the L1 and L2 would be possible and they would be congruent (Conklin and Carrol, 2019).

Thirdly, binomial predictability measured with cloze probability significantly predicted learners’ response time and accuracy, while the other two measures, i.e., association strength and transitional probability, did not show any significant effect.

As cloze probability was calculated as the proportion of learners’ right responses in the binomial completion task, directly indicating learners’ mastery of the target binomials, it is logical that cloze probability is effective in predicting learners’ accuracy and speed in processing the binomials.

The non-significant role of transitional probability combined with the significant role of cloze probability has important implications for the discrepant findings of Frisson et al. (2005) and McDonald and Shillcock (2003a, b) concerning the role and the relationship of these two variables in language processing. Specifically, McDonald and Shillcock (2003a, b) concluded from their studies that transitional probability operates independently from “regular” contextual predictability (i.e., cloze probability) effects. Later, Frisson et al. (2005) found that when cloze probability was well controlled, the effects of transitional probability disappeared while regular predictability effects were still observed. Therefore, they concluded that transitional probability as contingency statistics is most likely just one of the components influencing cloze probability. They also suggested that while it would be difficult or impossible to find transitional probability effects without cloze probability effects, it should be possible the other way around, which is supported by the present finding. Besides, the finding is in line with Arcara et al.’s (2012) that no significant effects were found for either the first or the second word transitional probability on L1 binomial processing. Additionally, in Carrol and Conklin’s (2020) eye-tracking study of L1 processing of collocations, idioms, and binomials, transitional probability was significantly correlated with cloze probability, and to avoid collinearity, it was removed from the analysis and only cloze probability was used as a measure of predictability. In short, the result of transitional probability of the present study and relevant studies in the literature points to its limited role in MWE processing, and as a measure of contingency, it is one of the factors that influence cloze probability.

The result of the non-significant role of association strength is to some extent in line with the finding in Siyanova-Chanturia et al. (2017) that binomials (e.g., *knife and fork*) had a processing advantage, but grammatically plausible, infrequent (novel) three-word combination whose two content words were as strongly associated (e.g., *spoon and fork*) did not. It shows the binomial status entails more than just two strongly associated words linked together by the conjunction *and* in that binomials are meaningful MWEs learners frequently encountered in the language input. The strong association between the two constituent words of a binomial is just one of its features. Carrol and Conklin (2020) also found that association strength made no or very limited contributions to the L1 processing of most phrase and word level reading patterns.

In all, the findings of the different roles of the three measures of binomial predictability call for studies to “pick and choose” appropriate indexes for a specific construct.

## 7 Conclusion

The present study was one of the few studies investigating L2 processing of English binomials. Through an acceptability judgment task, it revealed that Chinese EFL learners experienced difficulty in recognizing the formulaicity of binomial phrases. Moreover, their processing showed a congruency effect and the congruency effect further displayed an effect of the lexicalization of the congruent translation equivalents. Furthermore, binomial reversibility and predictability measured with cloze probability were found to be significant intralexical factors, while word and binomial frequency, and predictability measured with association strength and transitional probability did not play a significant role. The findings highlight the importance of distinguishing and examining different types of congruency in MWE processing. It also points to the fact that while binomials enjoy a formulaic advantage as other types of MWEs, their processing shows different patterns because of their unique distributional characteristics, especially their *A and B* configuration, reversibility, and close internal associations.

However, the present study is limited in several ways. First, the subjects only consist of L2 learners. Without the comparative data from the native speakers as a baseline, the uniqueness and typicality of the performance of the L2 speakers is to some extent open to question. Future studies can include native subjects, especially to test whether they show any congruency effect and L1-lexicalization effect in binomial processing as the L2 speakers did. If they do, then it would be rather interesting to explore the underlying reason, since this congruency effect or L1-lexicalization effect should theoretically only affect the L2 speakers, not the native speakers. Besides, the influence of intra-lexical factors on the L1 binomial processing is worth exploring as well. Secondly, as discussed earlier, for Chinese and English binomials, it is impossible to separate congruency from idiomaticity. Therefore, future studies are needed to examine similar questions with other languages, so that congruency can, at least to some extent, be separated from idiomaticity. For example, in Gyllstad and Wolter (2016), the congruent verb + noun collocations between English and Swedish can be completely literal or with the verbs being idiomatic and the nouns literal. Thirdly, because of the large number of variables, no interaction analysis was carried out with the data. It is recommended that future studies carry out research with a focus on the interactions of a manageable number of variables. For example, the reason for the significant role of congruency but not frequency found for L2 binomial processing might be that L1-L2 congruency takes precedence over frequency in the L2, considering that von Stutterheim et al. (2021) found motion event frames based on the L1 overrides frequency of occurrence of forms in the target language in L2 use. It would also be enlightening to explore the possible interactions between frequency and the three measures of predictability. Nonetheless, despite its limitations, the present study adds to our understanding of L2 binomial processing by revealing its similarities with and differences from the processing of other types of MWEs.

## Data Availability

The raw data supporting the conclusions of this article will be made available by the authors, without undue reservation.

## Appendix 1

**Table A1:** Sixty-four target binomials.

No.	Congruent binomials		Incongruent binomials	
	L1-lexicalized	L1-nonlexicalized		
1	Before and after	Boys and girls	Aches and pains	Loud and clear
2	Buy and sell	Doctors and nurses	Alive and well	Now and then
3	East and west	Family and friends	Back and forth	Odds and ends
4	Eat and drink	Health and safety	Bits and pieces	On and off
5	Gold and silver	How and why	Black and white	One and only
6	Good and bad	Name and address	Bread and butter	Over and above
7	High and low	Quick and easy	Come and go	Pick and choose
8	Hands and feet	Spoken and written	Day and night	Rise and fall
9	Life and death	Time and money	Each and every	Safe and sound
10	Long and short	Words and phrases	Far and wide	Said and done
11	Men and women	Mr and mrs	First and foremost	Sick and tired
12	Public and private	Mum and dad	Forgive and forget	Thick and thin
13	Right and wrong	Theory and practice	Give and take	This and that
14	Large and small	Oil and gas	Heart and soul	Trial and error
15	Wind and rain	Knowledge and skills	Here and there	Ups and downs
16	Read and write	Physical and mental	In and out	Wait and see

**Table A2:** Sixty-four baseline items.

No.	Baseline items			
1	Far and low	On and forth	Wind and error	Theory and friends
2	Good and thin	One and off	Boys and pains	Spoken and private
3	High and bad	How and then	Name and women	Life and mrs
4	Loud and wide	Now and why	Time and girls	Before and out
5	Safe and white	Back and there	Bits and death	Over and only
6	Sick and easy	Here and after	Odds and wrong	Each and that
7	Long and clear	First and every	Rise and money	This and foremost
8	Thick and well	Mr and dad	Hands and downs	Buy and done
9	Large and short	Men and west	Right and silver	Eat and see
10	Quick and tired	Mum and feet	Words and pieces	Read and take
11	Alive and small	Oil and rain	Aches and skills	Come and forget
12	Black and sound	Day and soul	Bread and safety	Give and go
13	Public and mental	Ups and gas	Heart and butter	Pick and write
14	Forgive and choose	East and ends	Trial and night	SAID and drink
15	In and above	Gold and fall	Family and nurses	Wait and sell
16	Physical and written	Knowledge and address	Health and practice	Doctors and phrases
